# Quantitative Evaluation of Hemolysis Under Excessive Static and Repeated Negative Pressure During Blood Handling

**DOI:** 10.7759/cureus.97883

**Published:** 2025-11-26

**Authors:** Masanobu Tsurumoto, Saki Mimoto, Aoi Sengoku, Akari Goto, Keisuke Hayashi

**Affiliations:** 1 Department of Clinical Engineering, Faculty of Health and Welfare, Tokushima Bunri University, Takamatsu, JPN

**Keywords:** blood collection, blood handling, extracorporeal circulation, free hemoglobin, hemolysis, medical device safety, negative pressure, red blood cell damage, vacuum-assisted sampling

## Abstract

Background: Hemolysis, defined as the rupture of red blood cells (RBCs) with the release of hemoglobin into plasma, is a common concern during blood collection and extracorporeal circulation. Although excessive negative pressure has often been implicated as a contributing factor, the extent to which static negative pressure alone induces hemolysis remains unclear. This study aimed to quantitatively evaluate hemolysis under excessive static and repeated negative pressure conditions to clarify their respective roles in red blood cell damage.

Methodology: Pooled bovine blood was used to ensure uniform baseline conditions across all protocols. Two experimental conditions were examined: (i) static negative pressure at approximately −713 to −720 mmHg for 30 and 60 minutes, and (ii) repeated negative pressure loading with ten cycles alternating between near-vacuum (approximately −690 mmHg) and atmospheric pressure. Free hemoglobin (fHb) concentrations were measured spectrophotometrically using a commercial assay kit. Results below the limit of detection (LoD = 0.03 g/dL) were considered non-quantifiable and marked as “<0.03”.

Results: Under static negative pressure, mean fHb levels remained low (30 min: 0.040 ± 0.0071 g/dL; 60 min: 0.044 ± 0.0055 g/dL; n = 5 each). Repeated loading produced comparable values (0.050 ± 0.000 g/dL; n = 5), with no evidence of cumulative hemolysis. A transient detection near the LoD at 30 minutes was not sustained at 60 minutes, suggesting minimal, short-lived mechanical stress. Overall, neither static nor repeated negative pressure exceeding −700 mmHg caused meaningful hemolysis progression.

Conclusions: The findings support the theoretical understanding that blood behaves as an incompressible fluid, equilibrating hydrostatic pressure across the red blood cell membrane to prevent rupture under static vacuum conditions. These results indicate that dynamic factors, such as shear stress and cavitation during rapid aspiration or pump operation, rather than static negative pressure, are the primary contributors to hemolysis. This study provides evidence-based guidance for safer blood collection practices and the design of medical devices, emphasizing control of dynamic stresses rather than avoidance of static negative pressure.

## Introduction

Hemolysis, defined as the rupture of red blood cells (RBCs) with the release of hemoglobin into plasma, is a frequent complication during extracorporeal circulation and blood collection. It may lead to anemia, increased plasma-free hemoglobin, and interference with laboratory testing, thereby affecting both patient outcomes and diagnostic accuracy. Clinically, one of the most relevant manifestations of hemolysis is pseudo-hyperkalemia, which can mislead diagnostic interpretation and treatment decisions, emphasizing the importance of preventing even mild hemolysis [1].

Among the proposed mechanisms, excessive negative pressure during blood handling has been cited as a potential contributor through mechanical stress on RBCs. Previous suction studies reported increased free hemoglobin with higher vacuum levels, suggesting possible RBC injury under aspirational conditions [2]. Similarly, clinical and flow-based investigations have highlighted hemolysis risks under combinations of negative pressure and shear stress encountered during procedures [3,4]. Preanalytic factors related to specimen handling have also been implicated in hemolysis occurrence, particularly under vacuum collection systems [5].

From a fluid-mechanical perspective, however, blood is essentially incompressible. Under static conditions, hydrostatic pressures equilibrate across the RBC membrane, making it unlikely that static negative pressure alone generates the differential stresses required for membrane rupture. Prior mechanistic work indicates that hemolysis attributed to negative pressure is more likely associated with dynamic factors, such as shear and cavitation, rather than with static pressure itself [6-8]. Cavitation occurs when local pressure falls below plasma vapor pressure, generating vapor bubbles whose collapse produces strong shear forces capable of damaging RBCs [9,10]. These effects are generally associated with rapid pressure fluctuations or turbulent flow rather than steady-state negative pressure. This conceptual difference between static and dynamic mechanisms has been described qualitatively in previous studies and is important for distinguishing static pressure-induced hemolysis from dynamic mechanisms.

Experimental data further suggest that static negative pressures up to approximately −720 mmHg do not induce significant hemolysis, challenging the assumption that vacuum exposure alone is sufficient to cause RBC destruction under sealed, static conditions [6,11]. Nonetheless, concerns persist in clinical practice regarding vacuum-based blood collection and aspiration procedures that transiently generate high negative pressure [12,13]. Computational and in vitro studies have also identified shear stress thresholds in circulatory systems and valve prostheses that correlate with mechanical hemolysis [14-16].

Prevention of hemolysis is essential for maintaining laboratory accuracy and patient safety [1,12,13]. Current design guidelines for blood collection and extracorporeal circulation primarily emphasize shear stress, flow velocity, and catheter gauge [14-16], while the independent effect of negative pressure remains insufficiently characterized [2-4,6-8]. Clarifying whether excessive static negative pressure alone contributes to hemolysis is therefore important for evidence-based device design and clinical protocols.

Objective of the study

The objective of this study was to quantitatively evaluate whether excessive static negative pressure alone, isolated from shear, flow, and cavitation, can induce measurable hemolysis under sealed conditions. Using pooled bovine blood, we assessed free hemoglobin changes during exposure to static and repeated negative pressures exceeding −700 mmHg to determine whether static vacuum loading alone contributes to hemolysis.

## Materials and methods

**Figure 1:**
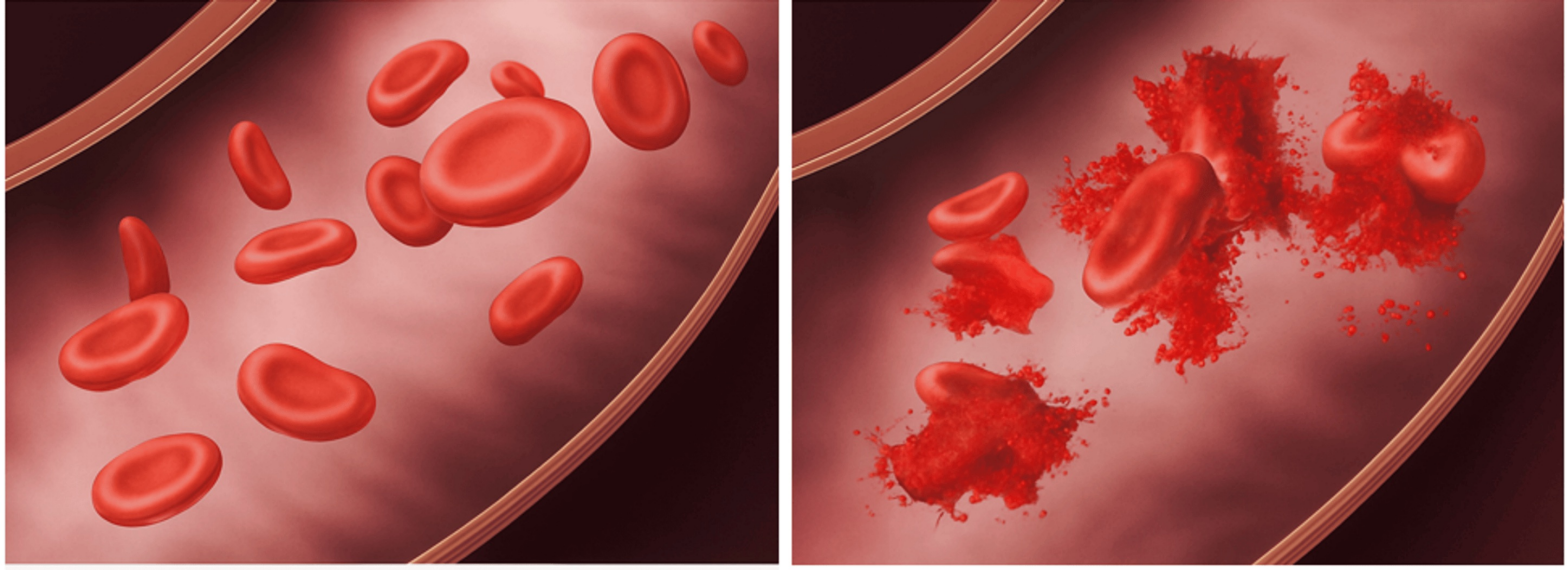
Dynamic-stress concept of hemolysis. Intact RBCs under steady static pressure (left) versus hemolysis under rapid pressure changes, shear/turbulence, and air–blood interface/cavitation (right). Illustration manually created by the authors using licensed Adobe Illustrator software.

Figure 1 conceptually illustrates the difference between static negative pressure and dynamic stress mechanisms (such as rapid pressure changes, shear/turbulence, and air-blood interface/cavitation) that can lead to hemolysis. 

Materials and sample collection

Bovine whole blood anticoagulated with sodium citrate was obtained from a certified supplier (Tokyo Shibaura Zoki Co., Ltd, Tokyo, Japan) for medical and medical-device research. Infectious screening was confirmed by the Tokyo Shibaura Meat Hygiene Inspection Office (Tokyo, Japan). Samples were stored at 4°C and used within 72 h to minimize pre-analytical hemolysis. For this study, a single pooled lot was gently mixed and aliquoted for all experimental conditions (static 30/60 min and repeated loading), thereby unifying the baseline across protocols. Prior to experimentation, blood aliquots were equilibrated at room temperature (23 ± 1°C) for approximately 30 minutes. The aspiration step for sample collection was performed using a standardized manual technique with a single, continuous draw to minimize shear variability. Hematocrit was measured prior to experimentation using the epoc Blood Analysis System (Siemens Healthineers, Erlangen, Germany). All syringes, collection tubes, and measurement equipment were sterile, medical-grade, and newly prepared.

Equipment and measurement tools

Static and cyclic negative pressure were applied using a Medrad VacLok syringe (Bayer Medrad, Warrendale, PA, USA). A digital handheld manometer (range: −750 to 750 mmHg) was connected via a T-connector to continuously monitor pressure inside the VacLok syringe. Whole blood was centrifuged at 3000 rpm for 10 min, plasma was collected, and free hemoglobin (fHb) was quantified using the Plasma/Low Hemoglobin kit (AMCO Inc., Tokyo, Japan). According to the manufacturer, the lower limit of detection (LoD) was 0.03 g/dL; values below LoD were treated as non-quantifiable and, for visualization, shown as “<0.03” with appropriate annotation. These values were excluded from numerical summary statistics. The Plasma/Low Hemoglobin kit (AMCO Inc.) and the digital manometer were operated in accordance with the manufacturers’ standard instructions as general medical and measurement devices. All procedures were performed under ambient laboratory conditions at 23 ± 1°C. All instruments used in this study are commercially available and do not require any special license or proprietary software.

Experimental protocols

**Figure 2:**
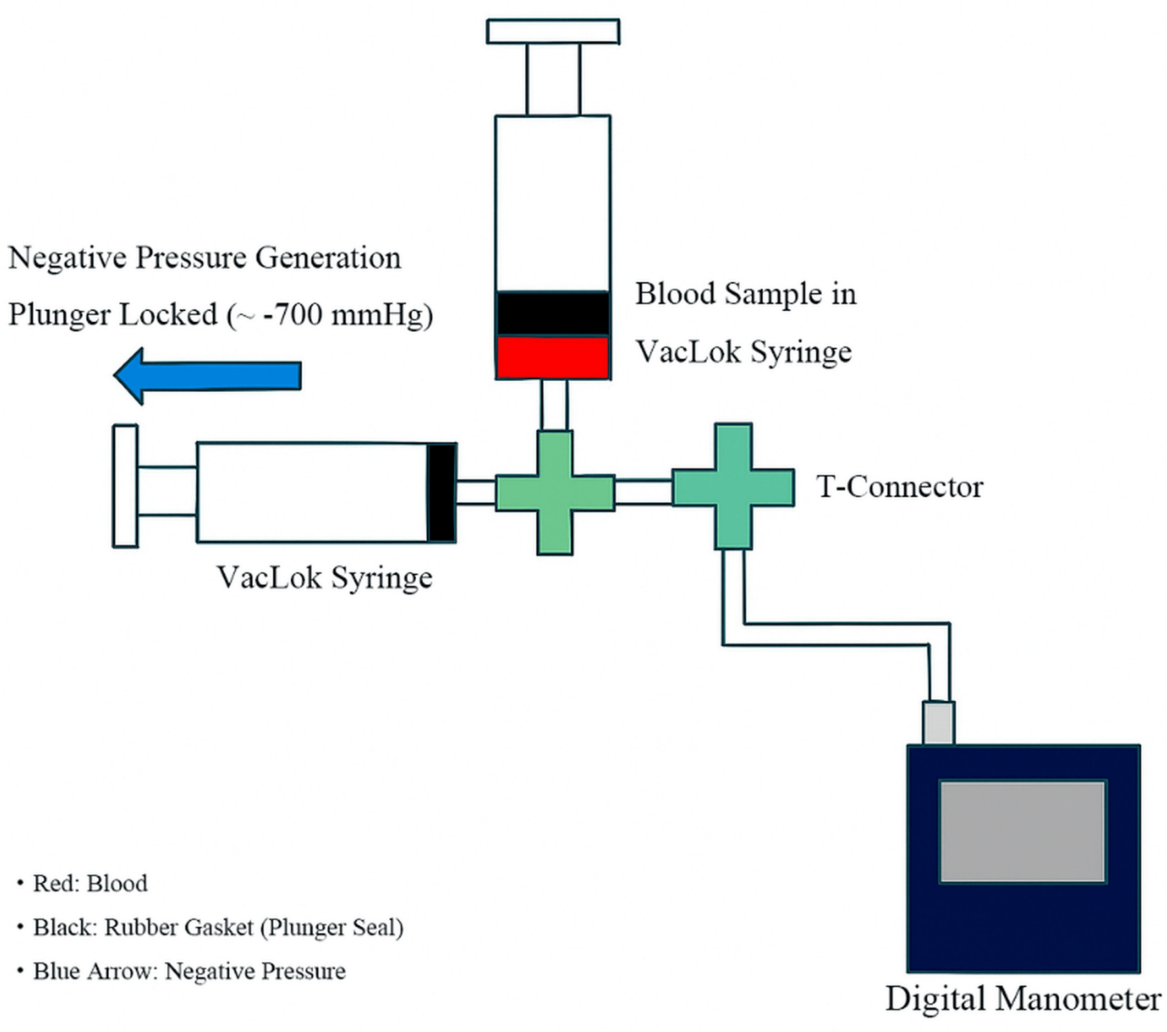
Experimental setup for generating and monitoring static negative pressure (∼−700 mmHg) using VacLok syringes and a digital manometer. A blood sample was aspirated into the VacLok syringe, and the plunger was locked to maintain negative pressure, while a T-connector enabled pressure monitoring. Blue arrow: direction of negative pressure; black: plunger seal; red: blood. Schematic diagram created by the authors.

The experimental setup for generating and monitoring static negative pressure is shown in Figure 2. Two protocols were used to investigate the effects of excessive static and repeated negative pressure on hemolysis progression, using aliquots from the same pooled blood.

Protocol 1: Static Negative Pressure (Time Course)

Approximately 5 mL of blood was aspirated into the VacLok syringe with minimal air entry. The plunger was pulled to ∼30 mL and locked to create a near-vacuum. The observed static pressure range was −713 to −720 mmHg at 23 ± 1°C under ambient laboratory conditions. The digital manometer was zero-calibrated prior to each experiment using a mercury reference at atmospheric pressure. Aliquots were sampled at baseline (control), 30 min, and 60 min. For each time point, n = 5 independent aliquots were measured; each aliquot was read in triplicate, and the triplicate mean was used for analysis.

Protocol 2: Repeated Negative Pressure Loading (10 Cycles)

Using the same setup, aliquots were subjected to ten cycles alternating between near-vacuum negative pressure and atmospheric pressure. Based on manometer recordings, the duration of each cycle was consistent and generally remained within approximately 15 seconds, without a predetermined dwell time at peak negative pressure. The peak negative pressure during cycling was approximately −690 mmHg, verified by the manometer. Again, n = 5 independent aliquots were tested; each aliquot was read in triplicate, and the triplicate mean was used for analysis. A baseline reference from the same pooled lot prior to negative-pressure exposure was used for contextual comparison.

Free Hemoglobin Quantification

Free hemoglobin was quantified using a dedicated analyzer (Plasma/Low Hemoglobin kit, AMCO Inc.), which automatically determines absorbance and displays the fHb concentration based on an internal calibration curve. Plasma samples were directly applied to the cuvette according to the manufacturer’s instructions, and measurement was performed at room temperature (23 ± 1°C). Each sample was measured in triplicate, and the mean value was used for analysis.

Data analysis

Unless otherwise specified, results are reported as mean ± standard deviation (SD) across aliquots, where n denotes the number of aliquots. Triplicate readings per aliquot were averaged before calculating summary statistics.

All data processing, descriptive statistics, and figure generation were performed using Python (version 3.11; libraries: NumPy, pandas, Matplotlib) and GraphPad Prism version 10.1 (GraphPad Software, San Diego, CA, USA) for cross-validation and graphical refinement.

The distribution of free hemoglobin (fHb) values was assessed using the Shapiro-Wilk test for normality. Given the small sample size (n = 5 aliquots per condition) and clustering of measurements near the assay’s lower limit of detection (LoD = 0.03 g/dL), analyses were primarily descriptive, and no formal hypothesis testing was conducted.

Values below the LoD were excluded from numerical summaries but are explicitly indicated in figures as “<0.03” with appropriate annotation. Figures display individual data points overlaid with mean ± SD to visualize variability and distribution within each condition.

## Results

Static negative pressure

**Figure 3:**
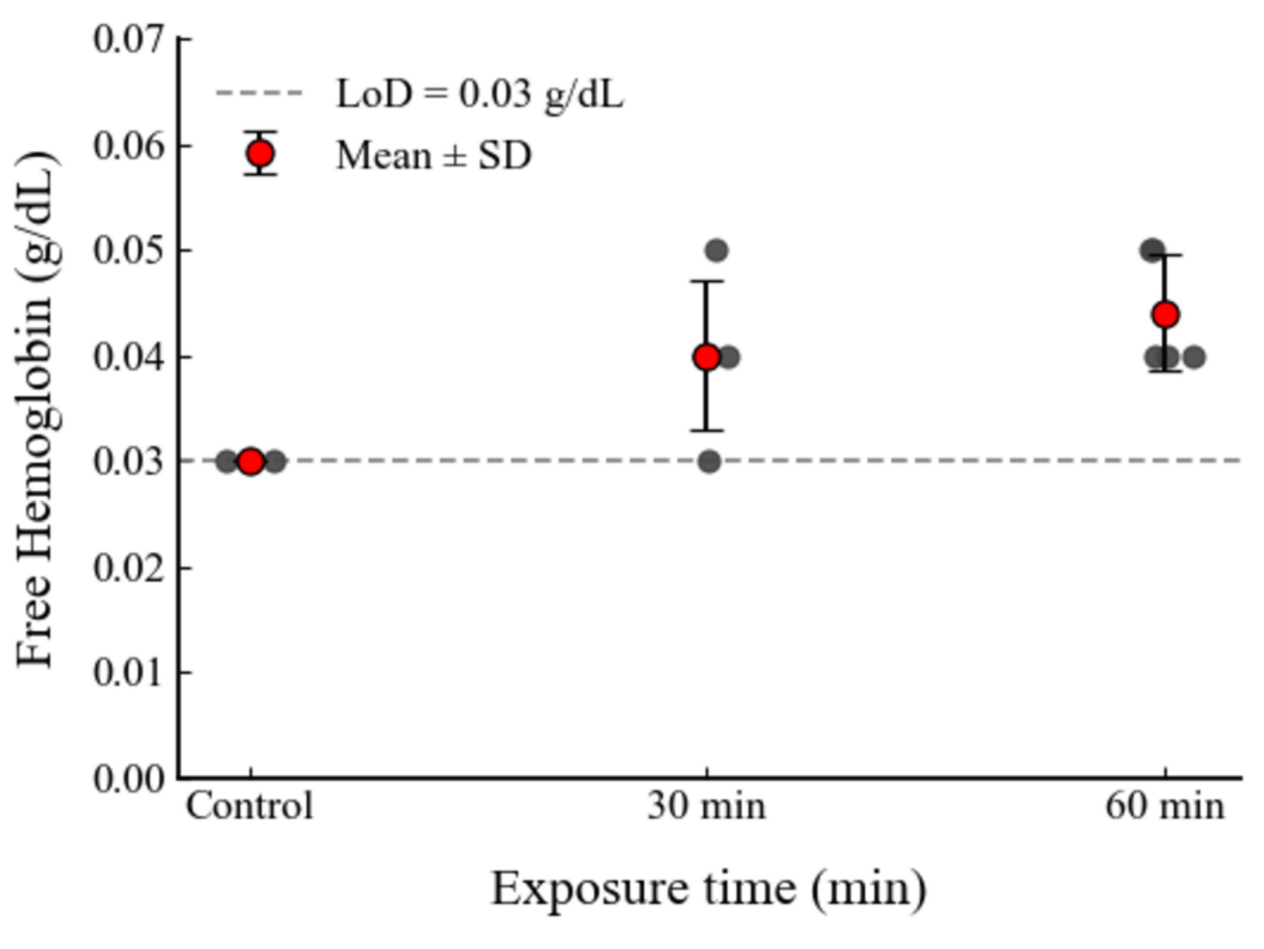
Free hemoglobin levels in pooled bovine blood samples exposed to static negative pressure (−713 to −720 mmHg) for 30 and 60 minutes. The control group represents baseline values obtained from two independent samples, each measured in triplicate. Each data point represents an independent aliquot (n = 5 for the 30-min and 60-min groups; n = 2 for the control), measured in triplicate. Results are expressed as mean ± standard deviation (SD). The dashed line indicates the assay’s lower limit of detection (LoD = 0.03 g/dL); values below this threshold are indicated as “<0.03”. The graph was generated by the authors from the experimental data.

Exposure to static negative pressure in the range of −713 to −720 mmHg was tested. At 30 minutes, the mean fHb was 0.040 ± 0.0071 g/dL (n = 5), and at 60 minutes it was 0.044 ± 0.0055 g/dL (n = 5). Both values were close to the baseline measurement (Figure 3).

Repeated negative pressure loading

**Figure 4:**
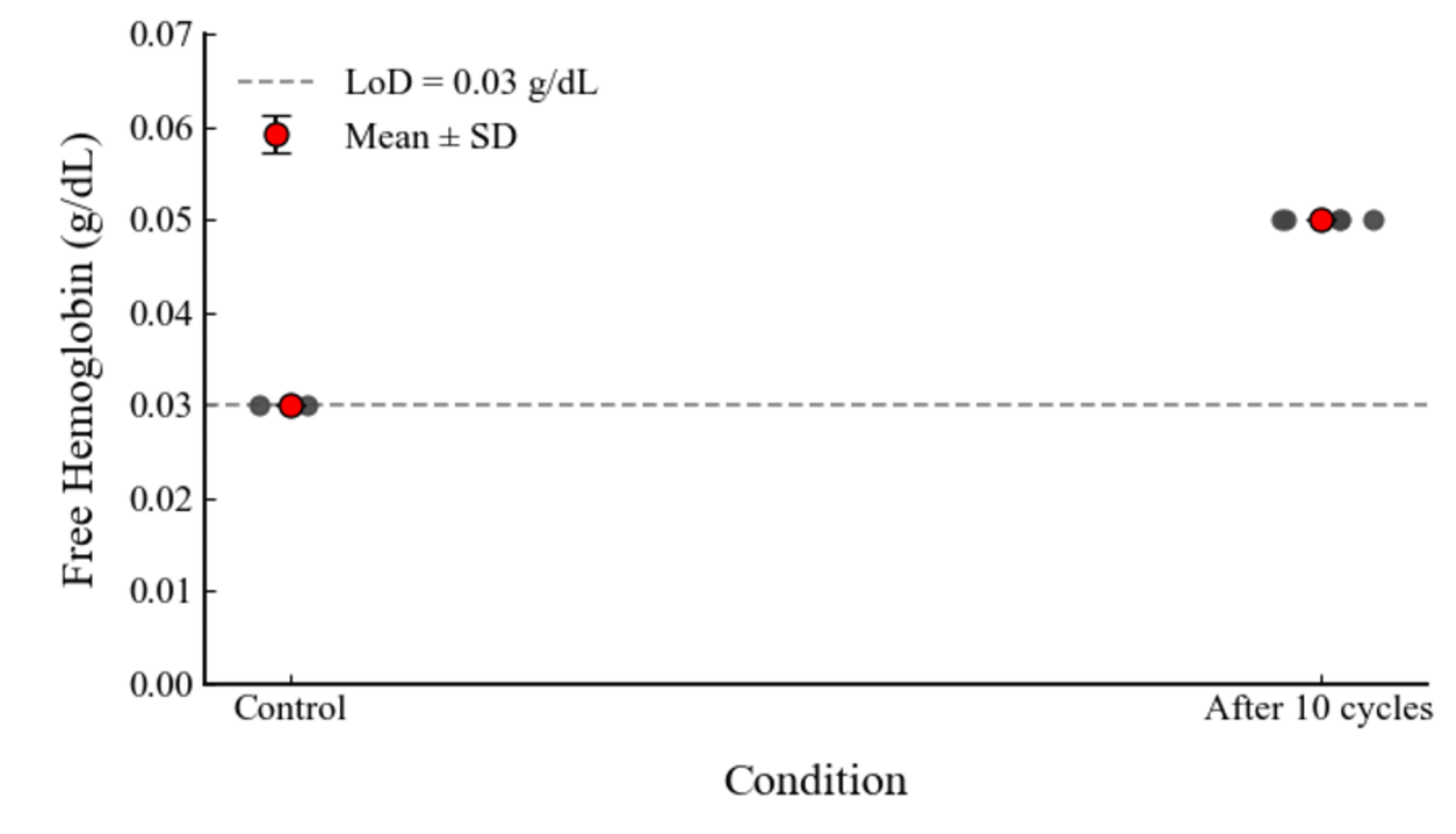
Free hemoglobin levels in pooled bovine blood samples before and after ten repeated negative-pressure cycles (approximately −690 mmHg). The control group represents baseline values obtained from two independent samples, each measured in triplicate. The post-loading group represents five independent aliquots, each measured in triplicate. Results are expressed as mean ± standard deviation (SD). Values below the assay’s lower limit of detection (LoD = 0.03 g/dL) are indicated as “<0.03”. The graph was generated by the authors from the experimental data.

Following repeated negative pressure loading with peak values at approximately −690 mmHg, all aliquots yielded the same fHb value of 0.050 g/dL after ten cycles (n = 5), resulting in a mean of 0.050 ± 0.000 g/dL. Across all tested conditions, the measured fHb values were ≤ 0.05 g/dL (Table 1, Figure 4).

**Table 1:** Free hemoglobin levels under static and repeated negative pressure conditions.

Condition	Pressure (mmHg)	Exposure time	n	Mean fHb (g/dL) ± SD
Control	0	—	2	<0.03
Static negative pressure	−713 to −720	30 min	5	0.040 ± 0.0071
Static negative pressure	−713 to −720	60 min	5	0.044 ± 0.0055
Repeated negative pressure	−690	10 cycles	5	0.050 ± 0.000

## Discussion

This study demonstrated that neither static negative pressure maintained at approximately −713 to −720 mmHg nor repeated negative pressure loading at approximately −690 mmHg produced meaningful hemolysis progression in bovine blood. Under static conditions, mean free hemoglobin values remained low at both 30 minutes (0.040 ± 0.0071 g/dL) and 60 minutes (0.044 ± 0.0055 g/dL; n = 5 each). Similarly, repeated loading over 10 cycles yielded uniformly low values (0.050 ± 0.000 g/dL; n = 5). These results indicate that excessive static or cyclic negative pressure, when applied in isolation, does not significantly elevate free hemoglobin concentrations.

**Figure 5:**
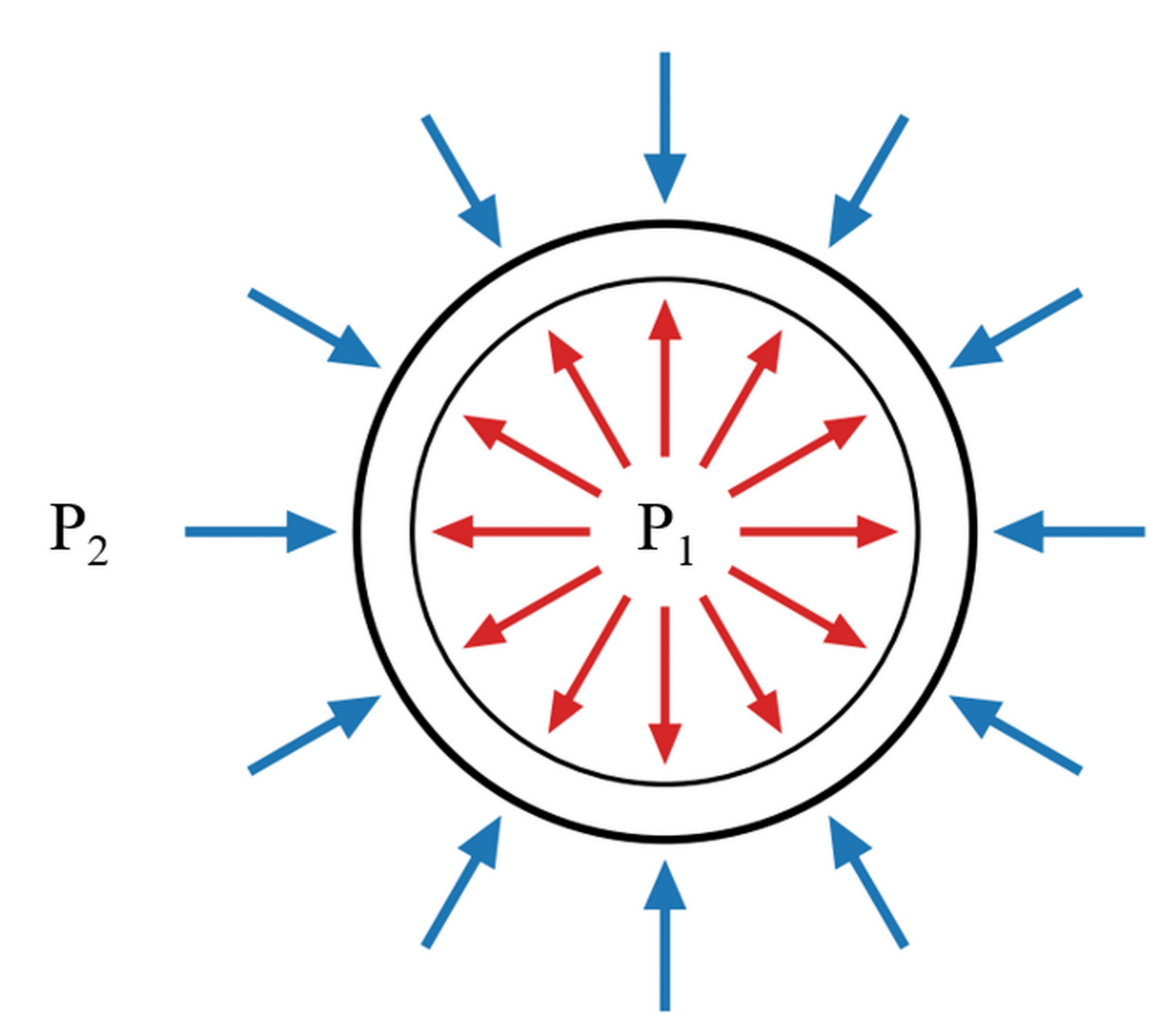
Static isobaric model under negative pressure. Equal hydrostatic pressure acts on both sides of the RBC membrane: P1 (cytoplasmic) and P2 (plasma) remain equal (P1 = P2), yielding no transmembrane gradient (ΔP = 0) and no deformation; thus, hemolysis does not occur under purely static conditions. Conceptual illustration created by the authors.

From a mechanistic standpoint, blood behaves as an incompressible fluid. Each red blood cell is suspended in plasma and surrounded by liquid both inside and outside the membrane. Therefore, when a static hydrostatic pressure is applied, the pressure is transmitted uniformly through these fluids, resulting in no pressure gradient or deformation across the membrane. Consequently, static negative pressure alone cannot generate the differential mechanical stress required to rupture red blood cells (Figure 5).

Chambers et al. observed no significant hemolysis up to −720 mmHg, and our findings extend this safety margin by confirming similar outcomes at pressures exceeding −700 mmHg [6]. In contrast, suction studies have reported hemolysis under rapid aspiration with increasing vacuum, and mechanistic and Couette-flow investigations emphasize shear and cavitation as dominant drivers of RBC damage rather than static pressure per se* *[5]. Dynamic phenomena such as bubble collapse and turbulent flow can generate localized high shear forces capable of disrupting RBC membranes [6-11]. Our results, therefore, support the view that static negative pressure alone is insufficient to cause hemolysis, whereas dynamic factors remain the primary concern.

Positioning and novelty

Prior evidence for tolerance to extreme negative pressure was predominantly obtained in flowing or suction systems, where shear and air-blood interfaces can confound interpretation. By employing a sealed, static system with continuous manometry, we experimentally separated hydrostatic loading from shear and interfacial phenomena. In addition, by testing repeated near-vacuum cycling (approximately −690 mmHg to atmospheric pressure, 10 cycles) without an air interface, we specifically examined whether pressure history alone promotes hemolysis. Under both conditions, fHb remained uniformly low, indicating that static or cyclic negative pressure by itself is not a dominant driver when shear and gas-liquid interfaces are minimized.

Methodological strengths

This study has several methodological strengths. The experimental system enabled isolation of static negative pressure from shear-related mechanisms, which is technically challenging in suction-based environments. Using a single pooled lot minimized baseline variability, and continuous manometry ensured that pressure levels were sustained throughout each protocol. Triplicate measurements improved measurement reliability, and direct comparison of static and cyclic loading conditions strengthened the interpretation of the findings.

Practical implications

Clinically, hemolysis is most often encountered under dynamic stress, such as rapid aspiration, inappropriate pump settings, or turbulent flow through small-bore catheters. Preventive strategies should therefore prioritize control of flow rates, needle/catheter gauge, aspiration technique, and circuit design to reduce shear and cavitation, rather than imposing strict caps on static vacuum. In the present sealed static model, negative pressures up to approximately −720 mmHg did not produce measurable hemolysis, suggesting that static vacuum within this range can be considered hemodynamically tolerable under shear-free conditions.

Limitations

Several limitations should be acknowledged. First, bovine blood was used as a surrogate due to ethical and practical constraints, and species differences in red blood cell membrane composition and fragility may limit extrapolation to human blood. Second, the exposure duration (60 minutes) and the number of negative pressure cycles (10) were limited, and microcavitation or transient shear during handling was not directly quantified. Moreover, gas quantification was not performed, and the sealed static design may not fully represent physiological gas-liquid interactions under dynamic flow conditions. Third, the assay detection limit (0.03 g/dL) precluded assessment of trace-level hemolysis below this threshold.

## Conclusions

This study demonstrated that excessive static negative pressure in the range of −713 to −720 mmHg and repeated loading at approximately −690 mmHg did not induce meaningful hemolysis progression in bovine blood under controlled conditions. Free hemoglobin levels remained low across all settings (30 min: 0.040 ± 0.0071 g/dL; 60 min: 0.044 ± 0.0055 g/dL; repeated loading: 0.050 ± 0.000 g/dL; n = 5 each), with no evidence of progressive or cumulative increase. These findings extend prior reports by confirming that even static pressures exceeding −700 mmHg do not significantly promote hemolysis, supporting the notion from previous studies that dynamic factors such as shear stress and cavitation are likely the primary drivers of red cell injury. Notably, by isolating static hydrostatic loading in a sealed system and additionally testing repeated near-vacuum cycling without an air interface under a unified baseline, we clarify that negative pressure per se (even when extreme or cycled) does not materially increase fHb, although gas-phase effects or microbubble formation were not directly assessed in this model.

Although this study was limited by the use of bovine blood, 60-minute maximum static exposure, and ten pressure cycles, the results highlight that preventive strategies to minimize hemolysis should primarily focus on controlling dynamic parameters-such as aspiration speed, circuit design, and needle/catheter gauge-, while recognizing that static negative pressure can indirectly influence flow-related shear through pressure gradients, rather than treating static vacuum as an isolated risk factor in blood collection or extracorporeal procedures.
